# Supplementary material for: BRCA1 Promoter Hypermethylation is Associated with Good Prognosis and Chemosensitivity in Triple-Negative Breast Cancer
Source: Cancers (Basel). 2020 Mar 30;12(4):828. doi: 10.3390/cancers12040828 (PMC7225997; doi:10.3390/cancers12040828)
Supplement: Supplementary file 1 [file cancers-12-00828-s001.pdf]

Table S1A. Multivariate analysis including BRCA1 IHC expression: Overall population (N=245)				
	RFS		OS	
	HR [95% CI]	*p*-value	HR [95% CI]	*p*-value
**Tumor size**				0.003
T1			1	
T2			2.00 [1.11; 3.58]	
T3/T4			4.27 [1.83; 9.95]	
**Nodal status**		<0.001		0.003
N-	1		1	
N+	5.01 [2.95; 8.53]		2.29 [1.34; 3.92]	
**Histology**		0.049		0.005
Ductal	1		1	
Lobular	1.11 [0.47; 2.62]		0.45 [0.15; 1.30]	
Other	0.30 [0.09; 0.97]		0.27 [0.09; 0.75]	
**Adjuvant chemotherapy**		<0.001		<0.001
No	1		1	
Yes	0.39 [0.23; 0.66]		0.30 [0.19; 0.49]	
**BRCA1 IHC expression**		0.368		0.544
BRCA1-/Equivocal	1		1	
BRCA1+	1.28 [0.74; 2.21]		1.17 [0.70; 1.95]	

Table S1B. Multivariate analysis including BRCA1 IHC expression: Adjuvant Chemotherapy = Yes (N=181)				
	RFS		OS	
	HR [95% CI]	*p*-value	HR [95% CI]	*p*-value
**Tumor size**				0.051
T1			1	
T2			2.62 [1.15; 5.95]	
T3/T4			2.05 [0.56; 7.47]	
**Nodal status**		<0.001		0.030
N-	1		1	
N+	3.61 [1.89; 6.90]		2.15 [1.07; 4.30]	
***BRCA1* Promoter Methylation**		0.031		0.060
No	1		1	
Yes	0.37 [0.13; 1.03]		0.40 [0.14; 1.16]	
**BRCA1 IHC expression**		0.100		0.272
BRCA1-/Equivocal	1		1	
BRCA1+	1.76 [0.87; 3.54]		1.49 [0.72; 3.06]	

Table S1C. Multivariate analysis including BRCA1 IHC expression: Adjuvant Chemotherapy = No (N=66)				
	RFS		OS	
	HR [95% CI]	*p*-value	HR [95% CI]	*p*-value
**Tumor size**				0.023
T1			1	
T2			1.34 [0.57; 3.13]	
T3/T4			5.04 [1.53; 16.6]	
**Nodal status**		<0.001		0.019
N-	1		1	
N+	6.79 [2.99; 15.4]		2.86 [1.21; 6.75]	
**Histology**				0.022
Ductal			1	
Lobular			0.30 [0.07; 1.38]	
Other			0.22 [0.05; 1.00]	
**BRCA1 IHC expression**		0.628		0.806
BRCA1-/Equivocal	1		1	
BRCA1+	0.80 [0.33; 1.93]		0.91 [0.41; 1.98]	

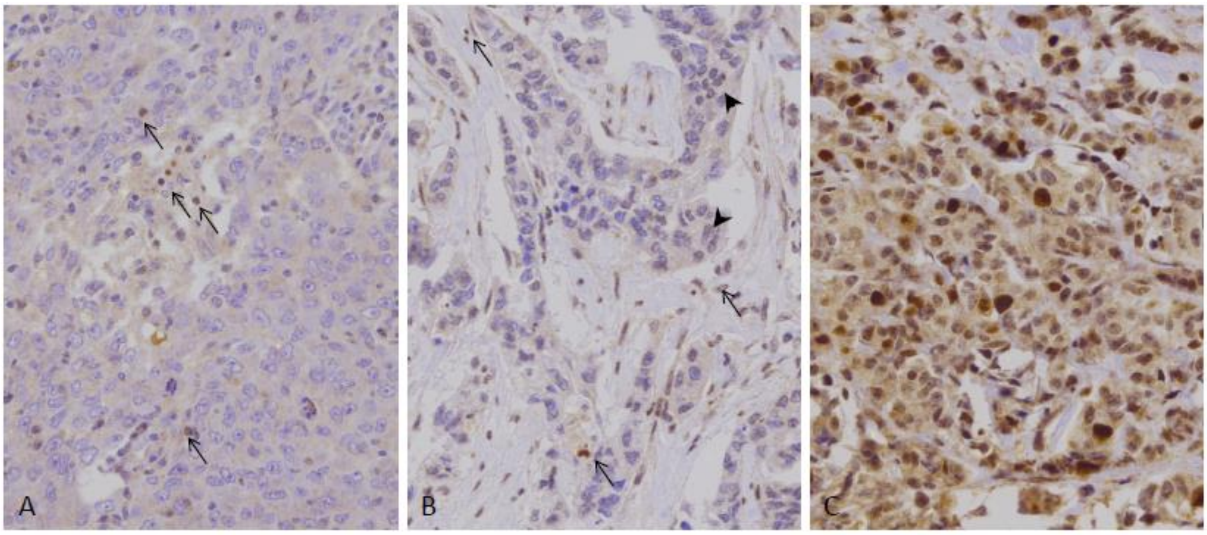

Figure S1: Examples of BRCA1-immunostained triple negative breast cancers. (**A**): loss of BRCA1 expression in tumor cells with the presence of positive internal controls (arrows); (**B**): equivocal BRCA1 expression with weak staining intensity of positive tumor cell nuclei (arrow heads) with the presence of positive internal controls (arrows); (**C**): retained BRCA1 expression with numerous strongly positive tumor cell nuclei. *Immunoperoxydase x400.*
